# Periodontitis in Patients Undergoing Coronary Angiography: A Cross-Sectional Study

**DOI:** 10.7759/cureus.75320

**Published:** 2024-12-08

**Authors:** Atanaska Nyagolova, Velislava Slavova, Radosveta Angelova, Rositsa Hristova, Dilyana Tonkova, Zlatina Tsoneva, Anzhela Bakhova, Stefan Peev, Svetoslav Georgiev

**Affiliations:** 1 Department of Periodontology and Dental Implantology, Medical University "Prof. Dr. Paraskev Stoyanov", Varna, BGR; 2 Department of Invasive Cardiology, University Hospital "St. Marina", Varna, BGR; 3 Department of Social Medicine and Healthcare Organisation, Medical University "Prof. Dr. Paraskev Stoyanov", Varna, BGR

**Keywords:** coronary artery disease, coronary obstruction, periodontal pathogens, periodontitis, syntax score

## Abstract

Background

Cardiovascular diseases (CVDs) are the leading cause of mortality worldwide, with coronary artery disease (CAD) being the primary contributor. Periodontitis, a common non-communicable disease, has been associated with an increased risk of CVD. Previous studies have suggested a link between the severity of periodontitis and the degree of coronary artery obstruction.

Objective

This study aims to investigate the correlation between the severity of periodontitis and coronary artery stenosis, as measured by the SYNTAX score I (Synergy Between Percutaneous Coronary Intervention with TAXUS and Cardiac Surgery), and to examine the association between severe periodontitis and other coronary pathologies, such as diffuse coronary disease and coronary thrombosis.

Materials and methods

An observational cross-sectional study was conducted at the Second Cardiology Clinic, University Multiprofile Hospital for Active Treatment “St. Marina”, Varna, Bulgaria, from December 2021 to January 2024. A total of 199 patients aged 45-64 years, indicated for coronary angiography, were included. Periodontal assessment included measuring probing pocket depth (PPD), clinical attachment loss (CAL), bleeding on probing (BoP), and plaque index (PI). Subgingival plaque samples were analyzed for periodontal pathogens. Coronary angiography was performed, and the SYNTAX score I was calculated to assess the severity of coronary artery stenosis. Statistical analyses, including chi-square tests and Spearman's correlation, were conducted to evaluate the associations.

Results

Among the 199 participants, 74.9% had severe periodontitis (stage III and IV). A weak but statistically significant correlation was found between mean CAL, periodontitis stage, and the SYNTAX score I (p < 0.01 and p < 0.05, respectively), indicating that more severe forms of periodontitis were associated with greater coronary artery stenosis. No correlation was observed between the presence of periodontal pathogens or the total microbial count and the SYNTAX score I. Additionally, no association was found between severe periodontitis and other coronary conditions, such as diffuse coronary disease and thrombosis.

Conclusion

This study provides new insights into the relationship between periodontal infection and coronary artery disease. Our findings suggest that severe periodontitis is significantly associated with a higher frequency and more complex coronary lesions, as indicated by the SYNTAX score I. However, no link was observed between specific periodontal pathogens and coronary stenosis. These results underscore the importance of early diagnosis and management of periodontal infections in patients with CAD, highlighting the potential benefits of an integrated approach to managing cardiovascular and periodontal health. Further studies are needed to explore these associations in more depth.

## Introduction

Cardiovascular diseases (CVDs) are a group of disorders affecting the heart and blood vessels and are the leading cause of mortality worldwide [1,2]. Coronary artery disease (CAD) is the primary cause of death from CVD globally [3]. In developed countries, CAD resulting from atherosclerosis (AS) of the coronary arteries leads to severe disability and causes more deaths than any other condition, including cancer [3]. In Europe, between one in five men and one in seven women die from CAD, and the disease accounts for 16% to 25% of all deaths in men [4].

Periodontitis is also a non-communicable disease (NCD) with a high global prevalence of 45-50%, with severe periodontitis affecting 11.2% of the world’s population, making it the sixth most common human disease [5]. Periodontitis is a multifactorial inflammatory disease caused by microbial dysbiosis that leads to the destruction of connective tissue and alveolar bone through an immune-inflammatory response to periodontal pathogens in the dental biofilm [6].

Several periodontal pathogens are associated with an increased risk of cardiovascular diseases in humans. A case-control study reported a higher prevalence of periodontal pathogens in subgingival plaque in patients with CAD compared to controls without CAD [7]. According to Bale et al. (2017), there is a causal relationship between periodontitis and the development of atherosclerosis in cases where periodontal disease is caused by "high-risk" periodontal pathogens (Aggregatibacter (A.) actinomycetemcomitans, Porphyromonas (P.) gingivalis, Tannerella (T.) forsythia, and Treponema denticola) [8].

Coronary artery disease has high morbidity and mortality rates, with atherosclerosis (AS) as its pathological basis. Periodontal treatment has been shown to improve endothelial function and reduce biomarkers like C-reactive protein (CRP) and interleukin 6 (IL-6), especially in cardiovascular patients. P. gingivalis and A. actinomycetemcomitans exacerbate AS by inducing endothelial dysfunction, oxidative stress, and procoagulant responses while also promoting platelet activation, lipid uptake, foam cell formation, and vascular calcification through mechanisms involving CD36, TLR2, and ERK1/2-RUNX2 pathways [7]. The further disruption of the immune balance by reducing regulatory T-cells and enhancing pro-inflammatory Th17 responses contributes to plaque instability. Additionally, P. gingivalis employs molecular mimicry with HSPs and manipulates the complement system, particularly C5a, amplifying its role in AS pathogenesis [6]. This interplay between periodontal pathogens, endothelial dysfunction, and lipoprotein retention underscores the significant role of periodontitis in atherosclerotic vascular disease (ASVD) pathogenesis, highlighting its potential as a therapeutic target.

A link has also been established between tooth loss, the presence of serum antibodies against P. gingivalis and A. actinomycetemcomitans, and coronary heart disease [9]. Several observational studies have reported an association between the severity of periodontitis and the degree of coronary obstruction, as visualized through angiography [10-13].

Joschi et al. (2022) investigated the relationship between myocardial damage following infarction and the severity of CAD, and periodontitis and found a positive correlation between levels of troponin I, SYNTAX score I (SSI; (Synergy Between Percutaneous Coronary Intervention with TAXUS and Cardiac Surgery)), periodontal inflamed surface area (PISA), mean values of probing pocket depths (mean-PPD) and clinical attachment loss (mean-CAL), and the bacterial load of P. gingivalis [14]. No such correlation was found for T. forsythia and A. actinomycetemcomitans.

Another study did not find a correlation between the presence of periodontitis and the value of the SSI [15].

Our study aimed to investigate the correlation between the severity of periodontitis and the severity of coronary obstruction, visualized angiographically and measured using SSI. Additionally, we examined whether there is an association between the presence of severe periodontitis and other types of coronary pathology, such as diffuse coronary disease and coronary thrombosis, which are also due to atherosclerosis.

## Materials and methods

This observational cross-sectional study took place in the Second Cardiology Clinic of the University Multiprofile Hospital for Active Treatment “St. Marina”, Varna, Bulgaria between December 2021 and January 2024 after gaining approval from the Institutional Ethics Committee of Medical University of Varna (Protocol N 108/25.11.2021).

Eligible patients were included in the study after signing informed consent. Criteria for eligibility included age between 45 and 64 years and indication for coronary angiography. The exclusion criteria were age under 45 years or over 64 years and absence of natural teeth.

The selected age range was chosen to capture the overlapping onset and progression of the conditions studied. The onset of periodontitis is generally around the age of 45, coinciding with the clinical manifestation of atherosclerosis in this demographic. The upper age limit of 64 was set to minimize the impact of additional systemic conditions that become more common in older individuals and might otherwise confound the study's findings.

To investigate the effect of the periodontitis stage on coronary obstruction with a statistical power of 80% for the chi-square test of association, we calculated the required minimum sample size to be 174 participants (https://www.statskingdom.com/sample_size_all.html).

During the research, 199 patients were included. Every patient underwent a standard clinical examination before hospitalization, and after an angiography, every patient was periodontally examined and had subgingival plaque samples collected. The angiography was inspected and SSI was calculated (https://syntaxscore.org/calculator/start.htm).

Periodontal status

A full periodontal assessment was carried out by measuring the gingival recession, PPD and CAL in millimetres, the presence of bleeding on probing (BoP), and dental plaque around six sites (disto-buccal, mid-buccal, mesio-buccal, disto-lingual, mid-lingual, and mesio-lingual) were recorded for each tooth; dental implants and retained roots were excluded. Based on these measurements, mean PPD, mean CAL, BoP index (Ainamo & Bay index) and PI (O’Leary index) were calculated [16]. The number of missing teeth was recorded. Periodontitis was diagnosed according to the 2017 classification [17].

Collecting plaque samples

Testing for periodontal pathogens was performed using the PET test (MIP Pharma GmbH, Blieskastel, Germany). After registration of the periodontal status, the five sites with the greatest PPD were chosen for plaque sample collection. A sterile absorbing paper point was inserted into the periodontal pocket and kept in place for 20 seconds according to test manufacturer instructions and then placed in a plastic container. The container and paper points are included in the test kit. The collected plaque samples were sent to MIP Pharma GmbH and the results of their processing were sent back to our institution.

Angiography and SYNTAX score

After local anaesthesia, a catheter was inserted into the vascular system. After reaching the coronary arteries, a contrast (Iomeron, Bracco S. p. A., Milan, Italy) was injected to visualize the coronary tree. Angiographic images were recorded and later evaluated, and the SYNTAX score was estimated (https://syntaxscore.org/calculator/start.htm).

The SYNTAX score is a grading tool used to assess the complexity and severity of coronary artery disease (CAD) based on angiographic findings. It takes into account the number, location, and severity of blockages within the coronary arteries, as well as specific lesion characteristics like length, tortuosity, and bifurcation involvement. The score is calculated based on a series of criteria for each coronary lesion that has a diameter reduction of 50% or more in vessels 1.5 mm or greater in diameter. Each lesion receives a weighted score, which is then summed for a total score.

Low SYNTAX score (0-22): Indicates simpler, less extensive coronary disease, often managed effectively with percutaneous coronary intervention (PCI).

Intermediate SYNTAX Score (23-32): Suggests more complex CAD, where treatment decisions are often balanced between PCI and coronary artery bypass grafting (CABG).

High SYNTAX Score (≥33): Reflects highly complex, extensive disease, typically favouring CABG over PCI due to potential benefits in long-term outcomes.

Statistical analysis

The collected data were analyzed with the statistical software JAMOVI (The jamovi project (2024). jamovi (Version 2.6.13) [Computer Software]. Retrieved from https://www.jamovi.org). The correlation between periodontal indicators and SSI was assessed by Spearman’s test. A chi-square association test was performed to see if there is an association between severe periodontitis and other types of coronary pathology due to atherosclerosis, as well as regression analysis for investigating the correlation between SSI and the indicators for periodontal disease. Missing data were minimal and handled using a listwise deletion approach. This method ensured that only complete cases were included in the analysis, maintaining the integrity of the dataset and the robustness of our findings.

## Results

The baseline characteristics of the sample are listed in Table 1.

**Table 1:** Baseline characteristics of the sample IQR – Interquartile Range; n – Number of patients; BMI – Body Mass Index; LDL-C – Low-Density Lipoprotein Cholesterol; HDL-C – High-Density Lipoprotein Cholesterol; ACS – Acute Coronary Syndrome; ST – ST-segment on electrocardiogram; LM – Left Main Coronary Artery; LAD – Left Anterior Descending Coronary Artery; RCx – Ramus Circumflexus of the Left Main Coronary Artery; RCA – Right Coronary Artery; SYNTAX - Synergy Between Percutaneous Coronary Intervention with TAXUS and Cardiac Surgery; BoP – Bleeding on Probing; PI – Plaque Index; Mean-PPD – Mean Probing Pocket Depth; Mean-CAL – Mean Clinical Attachment Loss; PET-test – Periodontal Pathogen test

	Sample (n = 199)
Gender, n (%)	
Male	132 (66.3)
Female	67 (33.7)
BMI kg/m^2^, median (IQR)	28.7 (25.8 – 31.8)
Hypertension, n (%)	179 (89.9)
Diabetes, n (%)	47 (23.6)
Smokers, n (%)	97 (48.7)
Lipid profile, mmol/L, median (IQR)	
Total cholesterol (reference values: < 5.2 mmol/L)	4.910 (4.015 – 5.935)
LDL-C (reference values: < 2.6 mmol/L)	2.860 (2.025 – 3.730)
HDL-C (reference values: > 1.0 mmol/L)	1.210 (1.000 – 1.420)
Triglycerides (reference values: < 1.7 mmol/L)	1.500 (1.035 – 2.375)
Indications for invasive diagnostic, n (%)	
Angina pectoris	96 (48.2)
ACS without ST elevation	50 (25.1)
ACS with ST elevation	37 (18.6)
Valve prosthesis	14 (7.0)
Valve prosthesis + angina pectoris	2 (1.0)
Angiographic findings	
Stenosis, n (%)	152 (76.4)
LM stenosis, max %, median (IQR)	0 (0 – 0)
LAD stenosis, max %, median (IQR)	20 (0 – 80)
RCx stenosis, max %, median (IQR)	0 (0 – 50)
RCA stenosis, max %, median (IQR)	0 (0 – 70)
SYNTAX score I, median (IQR)	5 (0 – 16)
Thrombosis, n (%)	35 (17.6)
Diffuse disease, n (%)	26 (13.1)
Atonic changes, n (%)	14 (7.0)
Slow flow, n (%)	7 (3.5)
Periodontal examination findings	
BoP (Ainamo & Bay) > 10%, n (%)	198 (99.5)
PI (O’Leary) > 10%, n (%)	197 (99.0)
Mean-PPD, mm, median (IQR)	4.50 (3.83 – 5.50)
Mean-CAL, mm, median (IQR)	-5.83 (-7.83 – -4.25)
Periodontal diagnosis, n (%)	
Gingivitis	2 (1.0)
Stage I	4 (2.0)
Stage II	44 (22.1)
Stage III	41 (20.6)
Stage IV	108 (54.3)
PET-test results, median (IQR)	
Aggregatibacter actinomycetemcomitans	0.0 (0.0 – 0.0)
Porphyromonas gingivalis	7.5 x 10^4^ (9.95 x 10^2^ – 4.2 x 10^5^)
Treponema denticola	1.5 x 10^5^ (2.55 x 10^4^ – 4.7 x 10^5^)
Total microbial count	6.6 x 10^9^ (1.2 x 10^9^ – 4.9 x 10^10^)

The chi-square test for association showed that more severe forms of periodontitis are associated with a more frequent occurrence of coronary artery stenosis (Table 2). 

**Table 2:** Association between the severity of periodontitis and the presence of coronary stenosis

Stage of periodontitis	Patents without stenosis	Patients with stenosis	Total	X^2^	df	p-value
No periodontitis	0	2	2	14.4	4	0.006
Stage I	2	2	4			
Stage II	12	32	44			
Stage III	17	24	41			
Stage IV	16	92	108			
Total	47	152	199			

The correlation analysis showed a weak but statistically significant correlation between mean-CAL, stage of periodontitis, and SSI (Table 3). 

**Table 3:** Correlation between mean-CAL and SYNTAX I, and between the stage of periodontitis and SYNTAX I CAL - Clinical Attachment Loss; SYNTAX - Synergy Between Percutaneous Coronary Intervention with TAXUS and Cardiac Surgery

Correlation between SYNTAX-I and	Spearman's rho	df	p-value
Mean-CAL, mm	-0.199	197	0.005
Stage of periodontitis	0.166	197	0.019

No correlation was found between the presence of periodontal pathogens or the total microbial count and SSI (Table 4). 

**Table 4:** Correlation between periodontal pathogens and SYNTAX I SYNTAX - Synergy Between Percutaneous Coronary Intervention with TAXUS and Cardiac Surgery

Correlation between SYNTAX I and	Spearman's rho	df	p-value
Aggregatibacter actinomycetemcomitans	0.036	197	0.612
Porphyromonas gingivalis	0.098	197	0.168
Treponema denticola	-0.006	197	0.935
Total microbial count	-0.074	197	0.300

No association was found between the presence of severe periodontitis and coronary thrombosis, and diffuse coronary disease in the sample (Tables 5, 6). 

**Table 5:** Association between the presence of severe periodontitis and the presence of coronary thrombosis

Severe periodontitis	Negative for thrombosis	Positive for thrombosis	Total	X2	df	p-value
Yes	122	27	149	0.116	1	0.733
No	42	8	50			
Total	164	35	199			

**Table 6:** Association between the presence of severe periodontitis and the presence of diffuse coronary disease

Severe periodontitis	Negative for diffuse coronary disease	Positive for diffuse coronary disease	Total	X^2^	df	p-value
Yes	127	22	149	1.51	1	0.219
No	46	4	50			
Total	173	26	199			

The results from the simple linear regression between SSI and the indicators for periodontal disease are shown in Table 7.

**Table 7:** Results from the simple linear regression analysis CAL - Clinical Attachment Loss; PPD - Probing Pocket Depth

	Mean-CAL	Mean-PPD	Severity of periodontitis
R	0.188	0.138	0.190
R²	0.0354	0.0191	0.0361
Adjusted R²	0.0305	0.0141	0.0312
F-statistic	7.23	3.84	7.37
p-value (Model)	0.008	0.051	0.007
Intercept	4.257	4.30	1.63
Model Coefficient	-0.866	1.14	2.49
p-value (Coefficient)	0.008	0.051	0.007
Standardized Estimate	-0.188	0.138	0.190
Shapiro-Wilk p-value	<0.001	<0.001	<0.001
Max Cook's Distance	0.151	0.261	0.0686

## Discussion

The aim of our study is to establish the relationship between the severity of periodontal infection and coronary artery disease. Among all 199 patients participating in the study, only 2 did not have periodontitis while 149 (74.9%) were affected by severe periodontitis (stage III and IV) (Table 1). These data are even more severe than the reports regarding the global prevalence of periodontitis [5]. The lack of data on the prevalence of periodontal diseases among the Bulgarian population does not allow for a local comparison of the results of this study. However, it is highly likely that the prevalence of periodontitis in Bulgaria is higher compared to global prevalence data.

Approximately two-thirds of the patients studied are men (Table 1). These data confirm the findings from epidemiological studies on the higher prevalence of cardiovascular disease (CVD) [4] among men, and male gender as a risk factor for developing CVD [18,19]. The study of BMI shows a trend toward obesity. Approximately 90% of the patients have concomitant arterial hypertension, 49% are smokers, and 24% have diabetes, which emphasizes the role of elevated blood pressure in the development of vascular damage [18,19]. The leading risk factors for CVD in the sample are male gender, obesity, and arterial hypertension (Table 1).

The most common coronary pathology in the sample is stenosis, followed by thrombosis. Other types of pathology are significantly rarer. We sought a correlation between the severity of periodontal parameters and the severity of coronary atherosclerosis, measured by SYNTAX score I. We found a significant but weak correlation between SSI severity, mean-CAL and severity of periodontitis (Table 3). We did not find a correlation between SYNTAX score I and the presence of periodontal pathogens. We also did not find a correlation between the total number of microorganisms and the severity of SSI (Table 4).

We found that more severe stages of periodontitis are associated with a more frequent presence of coronary artery stenosis, and the association is statistically significant (p < 0.01) (Table 2).

The obtained data confirm the results of other studies on the correlation between the severity of periodontitis and the severity of coronary atherosclerosis [10-13].

We examined the potential link between the presence of thrombosis and diffuse coronary disease, which are also consequences of atherosclerotic changes and severe periodontitis (Tables 5, 6). The results did not show an association between these conditions. However, this does not exclude the possibility of a connection between these diseases and periodontitis in general. To study such a potential association in more detail, a case-control study would be appropriate.

Limitations

This study has several limitations that should be considered when interpreting the results. First, as an observational cross-sectional study, the research design limits our ability to establish causation. While associations between periodontitis severity and coronary artery disease were identified, longitudinal studies would be required to confirm any causal relationships.

The study was conducted on patients from a single hospital in Bulgaria, which may restrict the generalizability of the findings to other populations. Additionally, the prevalence of periodontitis and coronary artery disease may differ in other demographic groups, impacting the applicability of the results more broadly.

Another limitation is the absence of a non-periodontitis control group, which restricts the strength of the findings. Including a control group without periodontitis or with minimal disease would provide a clearer contrast in outcomes and improve the reliability of the associations observed.

Although some common risk factors, such as hypertension and smoking, were adjusted for, other potential confounders, including socioeconomic status, diet, and genetic predispositions, were not accounted for and may have influenced the observed relationships.

The study also did not find an association between specific periodontal pathogens and coronary stenosis; however, the microbial analysis was limited to a few high-risk pathogens, potentially overlooking other bacteria or combinations of microorganisms that could also play a role in cardiovascular disease.

Furthermore, while the SYNTAX score is a valuable tool for assessing the complexity of coronary artery disease, it may not capture all nuances of coronary pathology. Additional scoring methods could provide a more comprehensive evaluation.

Lastly, some risk factors, such as smoking status, were subject to reporting bias, as they relied on patient self-reporting rather than objective measures.

Future directions

Further research through longitudinal and multi-centre studies is essential to better understand the causal pathways linking periodontitis and coronary artery disease (CAD). These studies should aim to assess how periodontal pathogen diversity impacts cardiovascular health and explore the biological mechanisms underlying this relationship. Incorporating broader-risk factor adjustments, such as socioeconomic status, dietary habits, and genetic predispositions, could provide a more comprehensive understanding of the interplay between periodontitis and CAD. Additionally, the use of other CAD scoring methods may offer deeper insights into the severity and progression of cardiovascular disease in relation to periodontal health.

## Conclusions

This study offers important insights into the relationship between periodontitis severity and the complexity of coronary artery disease (CAD), indicating that severe periodontal disease may be associated with a higher likelihood of coronary artery stenosis. While these findings support the potential significance of periodontal health in cardiovascular risk assessment, the study’s limitations, namely, its cross-sectional design, single-centre sample, and lack of a control group without periodontitis, underscore the need for cautious interpretation and further investigation to confirm these associations.

Further longitudinal and multi-centre studies are needed to clarify the causal pathways between periodontitis and CAD and to assess the impact of periodontal pathogen diversity on cardiovascular health. Expanding risk factor adjustment to include socioeconomic, dietary, and genetic factors, as well as utilizing additional CAD scoring methods, may enhance our understanding of these complex interactions. Overall, this study underscores the importance of considering periodontal health in cardiovascular assessments and encourages a more integrated approach to managing patients at risk for both periodontitis and CAD.
